# Existential Therapy within Palliative Care: Searching for Meaning

**DOI:** 10.1192/j.eurpsy.2024.157

**Published:** 2024-08-27

**Authors:** F. Cunha, I. Santos, N. Castro, R. Andrade, E. Almeida, J. Abreu, J. Martins, R. Vaz, S. Borges

**Affiliations:** Centro Hospitalar Tondela-Viseu, Viseu, Portugal

## Abstract

**Introduction:**

Irvin D. Yalom defines existential psychotherapy as a dynamic therapeutic approach that focuses on concerns rooted in existence with the four ultimate concerns being death, isolation, meaning in life, and freedom. Patients in advanced stages of cancer often experience elevated levels of psychological distress, encompassing conditions such as depression, anxiety, and a sense of spiritual hopelessness. Recently, interest in spiritual well-being has prompted a new wave of interventions that directly target this population, namely logotherapy and other existential interventions based on existential principles.

**Objectives:**

In this review, the primary focus was to comprehend the current evidence on the application of existential psychotherapy for individuals coping with advanced cancer and give an overview of the therapy approaches used.

**Methods:**

Narrative review of scientific literature using Pubmed search engine.

**Results:**

Terao and Satoh identified nine types of existential psychotherapies which were investigated using randomized controlled trials for patients with advanced cancer or in terminal care: Meaning-Centered Group Psychotherapy (MCGP), Individual Meaning-Centered Psychotherapy (IMCP), Meaning-Making intervention (MMi), Meaning of Life Intervention, Managing Cancer and Living Meaningfully (CALM), Hope Intervention, Cognitive and Existential Intervention, Dignity Therapy, and Life-Review Interviews. All deal with the issues pointed by Yalom. Existential or spiritual well-being improvements were validated in MCGP, IMCP, Meaning of Life intervention, and Life-Review intervention.

**Conclusions:**

Current evidence is still based on a very limited number of studies. Additional research is needed to delve into the impact of existential psychotherapy on individuals facing advanced cancer.

**Disclosure of Interest:**

None Declared

